# MicroRNA miR-320a and miR-140 inhibit mink enteritis virus infection by repression of its receptor, feline transferrin receptor

**DOI:** 10.1186/s12985-014-0210-3

**Published:** 2014-12-03

**Authors:** Jia-zeng Sun, Jigui Wang, Shuang Wang, Daoli Yuan, Zhili Li, Bao Yi, Qiang Hou, Yaping Mao, Weiquan Liu

**Affiliations:** State Key Laboratory of Agrobiotechnology, Department of Biochemistry and Molecular Biology, College of Biological Sciences, China Agricultural University, No. 2 Yuanmingyuan West Road, Haidian District, Beijing, 100193 China

**Keywords:** Mink enteritis virus (MEV), miR-320a, miR-140, Transferrin receptor (TfR)

## Abstract

Mink enteritis virus (MEV) is one of the most important pathogens in the mink industry. Recent studies have shed light into the role of microRNAs (miRNAs), small noncoding RNAs of length ranging from 18–23 nucleotides (nt), as critical modulators in the host-pathogen interaction networks. We previously showed that miRNA miR-181b can inhibit MEV replication by repression of viral non-structural protein 1 expression. Here, we report that two other miRNAs (miR-320a and miR-140) inhibit MEV entry into feline kidney (F81) cells by downregulating its receptor, transferrin receptor (TfR), by targeting the 3′ untranslated region (UTR) of TfR mRNA, while being themselves upregulated.

## Introduction

Mink enteritis virus (MEV) is an autonomous parvovirus that causes important disease in mink, leading to huge economic losses worldwide. MEV is a single-stranded negative sense DNA virus belonging to the family *Parvoviridae*, with a genome of about 5 kb containing 2 main open reading frames (ORFs). MEV, a variant of feline panleukopenia virus (FPV) and highly homologous with canine parvovirus (CPV), causes a highly infectious acute disease of mink characterized by extensive virus replication in mesenteric lymph nodes and intestinal crypt epithelial cells, with an associated loss of intestinal mucosa, diarrhea, and a high rate of morbidity and mortality [1-5].

MicroRNAs (miRNAs) are endogenous and highly conserved small noncoding RNAs of length 18–23 nucleotides (nt), which have gained widespread attention as critical modulators in many biological processes including cell proliferation and differentiation, development and apoptosis. In animals, miRNAs are imprecisely complimentary to their mRNA targets and act by repression of target gene expression [6-9].

Attention has also been paid to the role of miRNAs as effectors in host-virus interaction networks [10,11], either by targeting cellular factors useful for virus replication [12,13] or by directly targeting virus mRNAs [14-17]. We have recently reported that a cellular miRNA miR-181b inhibits MEV infection by repression of viral non-structural protein 1 expression [18], which indicates that cellular miRNAs may play a direct role on viral mRNAs themselves.

For many animal viruses, cell entry and infection are initiated by receptor-mediated endocytosis involving specific cellular surface components. Transferrin receptor (TfR), is required for the import of iron into the cell, and is regulated by intracellular iron concentration. It has also been reported to be a receptor for MEV, controlling the first step in the viral infection process. TfR can be considered a model for the endocytosis and recycling of receptor-ligand complexes: it is excluded from lipid raft domains in the plasma membrane and is taken up rapidly from the cell surface via clathrin-mediated endocytosis [1,19-24]. Since TfR plays an important role in MEV infection, we therefore investigated whether miRNAs participate in the host-virus interaction by modulating its activity.

## Materials and methods

### Cell culture and MEV infection

Feline F81 cells, obtained from the American Type Culture Collection (ATCC), were cultured as monolayers in minimum essential medium (MEM) (Gibco, CA) containing 10% FBS (Hyclone, Logan, UT), and 1% penicillin-streptomycin (Gibco) at 37°C in a 5% CO_2_ atmosphere. MEV strain L was originally isolated from an infected animal in a mink farm, Liaoning province, China. The whole viral genome has been sequenced in our laboratory and found to have high identity with MEV strain Abashiri (GenBank accession, D00765.1). For infection, F81 cell monolayers were first dispersed by 0.25% trypsin and virus was added to the suspension before incubation in the original plates.

### Deep sequencing of small RNAs and analysis of the sequencing data [25]

F81 cells cultured in 6-well plates (Costar) were infected with MEV at an input multiplicity (MOI) of 1 pfu/cell. Uninfected cells were maintained as a control. Twenty-four h later, the triplicate cultures were pooled, total RNA was extracted by Trizol reagent (Invitrogen) and small RNAs with length of 18–30 nt were separated by PAGE. Ten μg samples of the isolated RNAs were submitted to Solexa (Illumina) for sequencing as cDNA libraries. Duplicate sequences were eliminated from the initial data set. The resulting sets of unique reads were mapped onto the feline genome [26,27] using the program Short Oligonucleotide Analysis Package (SOAP) [28]. Perfectly matched reads were also mapped onto the miRNAs of six reference species (*Homo sapiens*, *Canis familiaris*, *Mus musculus*, *Rattus norvegicus*, *Bos taurus* and *Sus scrofa*) listed in the Sanger miRBase (Release 18) using the Patscan tool [29] to identify homologs of known miRNAs.

### Prediction of miRNA targets in feline TfR mRNA 3′UTR

RNAhybrid tools (http://bibiserv.techfak.uni-bielefeld.de/rnahybrid/submission.html/) [30] were used to predict miRNA targets in TfR mRNA 3′UTR following the rules of no mismatch and G/U complementarity in miRNA seed sequences. RegRNA tools (http://vita.mbc.nctu.edu.tw/) [31] were also used to predict regulatory RNA motifs in the TfR mRNA 3′UTR. TargetScan (http://www.targetscan.org/) tools were used to predict conservative miRNA targets in the TfR mRNA 3′UTRs of different species.

### Plasmid construction

The luciferase expression vector pGL3-control (Promega) was used for construction of predicted miRNA candidate targets containing a luciferase reporter gene, with pRL-TK (Promega) as control. Sequences containing part of the TfR 3′UTR and candidate targets of miRNAs were amplified by RT-PCR and directionally inserted into the 3′UTR of the luciferase gene in the pGL3-control vector, generating pGL3-TfR 3′UTR. To facilitate cloning, the first PCR product amplified by the first pair of primers (5′-ATGTGGTACCTATACTTATATGAGAAC-3′ and 5′-TCCGTGTTCAAGCATTTTATTAAATC-3′) was used as a template and an *Xba* I restriction site (italics) was added to the 5’- (5′-GC*TCTAGA*ATGTGGTACCTATACTTATATGAGAACAGC-3′) and 3′- (5′-GC*TCTAGA*TCCGTGTTCAAGCATTTTATTAAATCAG-3′) secondary pair of primers. To further ascertain that the binding sites of the predicted miRNAs in TfR 3′UTR indeed existed, tetranucleotide mutations were generated in the two potential target sites of pGL3-TfR 3′UTR using PCR, resulting in mut320a-pGL3-TfR 3′UTR (5′-CACTAGATTTCTTTAGGCAGCA*CGAA*TTAATACAGGGTAGGTAC-3′ and 5′-GTACCTACCCTGTATTAA*TTCG*TGCTGCCTAAAGAAATCTAGTG-3′) and mut140-pGL3-TfR 3′UTR (5′-CTTCAAGTTAAAGTGAATAA*GGTG*TTAAAAATGTTCATGATAGAATC-3′ and 5′-GATTCTATCATGAACATTTTTAA*CACC*TTATTCACTTTAACTTGAAG-3′). Mutant plasmids were generated by PCR using PrimeSTAR MAX DNA Polymerase (TaKaRa), 50 ng of the parent vectors as templates and the complementary primers under the following conditions: 98°C for 3 min, followed by 30 cycles of 98°C for 10 s, 55°C for 15 s and 72°C for 90 s, followed by 72°C 10 min. The resulting products were digested with 1 μl *Dpn*-1 for 1 h at 37°C to remove the parental DNA. The remaining DNA was used to transform competent DH5α cells, and a number of colonies containing mutant plasmids were obtained and confirmed by sequencing (Shanghai Sangong Co.).

### miRNA mimics and inhibitors

The miR-320a, miR-320b, miR-140, miR-145, miR-152, miR-182 and miR-194 mimics and inhibitors, mut miR-320 mimics (in which the tetranucleotide mutation was complementary to mut320 pGL3-TfR 3′UTR) and mut miR-140 mimics (in which the tetranucleotide mutation was complementary to mut140 pGL3-TfR 3′UTR) were synthesized by GenePharma, Shanghai. All mimics were double-stranded RNA oligos, while inhibitors were single-stranded. Negative control mimics and inhibitors were also synthesized for control experiments. The inhibitors were modified by 2′-O-methylation. All sequences of mimics and inhibitors are listed below (italic letters are mutated bases):

miR-320a mimics: 5′-AAAAGCUGGGUUGAGAGGGCGA-3′

miR-320a inhibitors: 5′-UCGCCCUCUCAACCCAGCUUUU-3′

miR-320b mimics: 5′-AAAAGCUGGGUUGAGAGGGCAA-3′

miR-320b inhibitors: 5′-UUGCCCUCUCAACCCAGCUUUU-3′

miR-140 mimics: 5′-CAGUGGUUUUACCCUAUGGUAG-3′

miR-140 inhibitors: 5′-CUACCAUAGGGUAAAACCACUG-3′

miR-145 mimics: 5′-GUCCAGUUUUCCCAGGAAUCCCU-3′

miR-145 inhibitors: 5′-AGGGAUUCCUGGGAAAACUGGAC-3′

miR-152 mimics: 5′-UCAGUGCAUGACAGAACUUGG-3′

miR-152 inhibitors: 5′-CCAAGUUCUGUCAUGCACUGA-3′

miR-182 mimics: 5′-UUUGGCAAUGGUAGAACUCACACU-3′

miR-182 inhibitors: 5′-AGUGUGAGUUCUACCAUUGCCAAA-3′

miR-194 mimics: 5′-UGUAACAGCAACUCCAUGUGGA-3′

miR-194 inhibitors: 5′-UCCACAUGGAGUUGCUGUUACA-3′

Mut miR-320a mimics: 5′-AA*UUCG*UGGGUUGAGAGGGCGA-3′

Mut miR-140 mimics: 5′-CA*CACC*UUUUACCCUAUGGUAG-3′

Negative control (NC) mimics: 5′-UUCUCCGAACGUGUCACGUTT-3′

Negative control (NC) inhibitors: 5′-CAGUACUUUUGUGUAGUACAA-3′

### Transfection

To screen for selected miRNAs to downregulate the expression of TfR, F81 cells, at a confluence of 60-70% in 24-well plates (Costar), were transfected with mimics or inhibitors (10 nM) using Lipofectamine 2000 transfection reagent (Invitrogen). NC mimics and inhibitors were used as controls. To further determine whether the screened miRNAs could modulate the expression of TfR, F81 cells were transfected with the mimics in a dose-dependent manner. The cells were collected for TfR mRNA qPCR assay at 36 h post-transfection and western blot analysis and flow cytometry assay at 48 h.

To determine whether the selected miRNAs play a direct role in repression of luciferase expression from pGL3-TfR 3′UTR, 24-well plates seeded with F81 cells at 60-70% confluence were co-transfected with a mixture of pGL3-TfR 3′UTR (4 μg/ml) and pRL-TK vector (4 μg/ml) together with mimics (10 nM). The mut320a or mut140 pGL3-TfR 3′UTR and pRL-TK together with mimics (10 nM) were co-transfected to verify accuracy of the seed sequence. NC mimics were used as negative controls. After 36 h, the cells were harvested for relative luciferase activity assay.

To determine the effects of the selected miRNAs on MEV infection, F81 cells, at a confluence of 60-70% in 24-well plates, were transfected with mimics (10 nM). After 48 h, the cells were dispersed with 0.25% trypsin and infected with MEV (MOI = 0.1). Virus infection was measured by qPCR and flow cytometric analysis at the indicated times.

### Luciferase assays

To quantify the relative luciferase activity, a dual-luciferase reporter assay system kit (Promega) was used according to the manufacturer’s protocol. Co-transfected cells with a mixture of luciferase reporter plasmids were washed with cold phosphate-buffered saline (PBS). Passive lysis buffer (Promega: 100 μl) was then added to the cells in each well. After 10 min, the supernatants were clarified by centrifugation at 12,000 g for 30 s, and the luciferase activity was measured using a Modulus single-tube multimode reader (Promega). Relative luciferase expression was calculated as the expression of firefly luciferase (pGL3-control vector) divided by that of Renilla luciferase (pRL-TK).

### Real-time quantitative PCR (qPCR) analysis

To detect whether selected miRNAs can downregulate the expression of TfR, qPCR analysis was performed. After transfection of F81 cells with miRNA mimics or inhibitors, NC mimics or inhibitors as controls, total RNA was extracted and digested with DNase I (Takara). Two μg total RNA of each sample was reverse transcribed using M-MLV reverse transcriptase (Promega) according to the manufacturer’s protocol. The β-actin mRNA level was measured as a control. Primers used for amplification were: β-actin, 5′-CGGGACCTGACGGACTACCT-3′ and 5′-GGCCATCTCCTGCTCAAAAT-3′ and TfR, 5′-ATGATTGGCTACTTGGGCTATTG-3′ and 5′-CCTGATGGTGCTGGTGAACTC-3′.

To detect the viral genomic DNA quantitative level in F81 cells, total DNA was extracted and the concentration was measured. PCR amplication of a fragment of viral genomic DNA (5′-GCTTACGCTGCTTATCTTCGC-3′, 5′-TAATGTCCTATTTTCCCCCCC-3′) was performed.

To determine the expression level of miRNAs in F81 cells, total RNA was extracted, and 2 μg was polyadenylated using *E. coli* poly (A) polymerase according to the manufacturer’s protocol (Promega). The poly (A) reaction product was then reverse transcribed using M-MLV reverse transcriptase (Promega) and an adaptor primer [32] (5′-GCGAGCACAGAATTAATACGACTCACTATAGGTTTTTTTTTTTTVN-3′) according to the manufacturer’s protocol. PCR amplication was carried out using the specific miRNAs primers (miR-320a, 5′-AAAAGCGGGGAGAGGGCG-3′ and 5′-GCGAGCACAGAATTAATACGACTCAC-3′ and miR-140, 5′-CAGTGGTTTTACCCTATGGTAGAAA-3′ and 5′-GCGAGCACAGAATTAATACGACTCAC-3′). U6 small RNA expression level was measured as a control using primers 5′-CTCGCTTCGGCAGCACA-3′ and 5′-AACGCTTCACGAATTTGCGT-3′. Cycling conditions for qPCR using FastSYBR Mixture (CWBIO) and the ViiA™ 7 real-time PCR System (Applied Biosystems) were 95°C for 20 s, followed by 35 cycles of 95°C for 3 s and 60°C for 30 s. The data were analyzed by the ΔΔCt method [33].

### Western blot assay

F81 cells transfected with mimics in a 24-well plate were washed 3 times with cold PBS, a mixture of 100 μl RIPA lysis buffer (HX-BIO) and 0.5 mM PSMF was added and the cells were harvested into Eppendorf tubes. After 30 min on ice and centrifugation at 12,000 g for 30 min, 25 μl supernatant was mixed with 25 μl each 2 × SDS sample buffer and boiled for 5 min. Samples were subjected to 10% SDS-PAGE gel and transferred to a nitrocellulose membrane (PALL Life Science). The membranes were blocked with 5% nonfat dry milk for 1 h, then incubated for 1 h at room temperature with purified primary mouse antibody CD71 (H68.4) (Santa Cruz: 1:500 dilution) or anti-β-actin antibody (MBL: 1:1,000 dilution) in nonfat milk. After 3 washes with Tris-buffered saline containing 0.05% Tween-20 (TBST), the membranes were incubated for 1 h at ambient temperature with the appropriate horseradish peroxidase-conjugated secondary antibody (MBL: 1:5,000 dilution) in TBST. Protein bands were visualized using ECL western blot substrate (Thermo), with β-actin as a control.

### Flow cytometry

Treated F81 cell monolayers were dispersed with 0.25% trypsin, harvested and fixed in 4% paraformaldehyde. After 3 washes with PBS and incubation for 1 h at 37°C with anti-CD71 mouse antibody (1:2500) or anti-MEV rabbit polyclonal antibody (prepared in this laboratory) at 1:100, the cells were washed 3 times with PBS, incubated with fluorescein isothiocyanate (FITC)-conjugated goat anti-mouse or anti-rabbit IgG antibody (MBL: 1:100 dilution) for 1 h at 37°C, washed another 3 times with PBS and analysed by BD FACSCalibur flow cytometry. Nonspecific rabbit polyclonal antibody (iso) (prepared in this laboratory) was used as an isotype control. The data were analyzed using BD CellQuest software.

### Argonaute 2 (Ago2) co-immunoprecipitation

Human anti-Ago2 antibody (Abnova) was first bound to protein A/G-Agarose (Abmart) in PBS for 30 min at 4°C. Treated F81 cells were harvested, washed and solubilized in RIPA lysis buffer (HX-BIO) and PSMF for 30 min on ice, then centrifuged at 12,000 g for 30 min to clarify the supernatant. The latter was then added to the Ago2/Agarose conjugate and incubated for 4 h at 4°C. Incubation of the supernatant with normal mouse IgG (MBL) was used as a negative control. RNA bound to the Ago2 protein was dissociated with Trizol reagent and reverse transcribed. TfR, miR-320a and miR-140 were quantified by qPCR analysis, with β-actin and U6 small RNA as internal controls.

### Statistical analysis

Data were analysed statistically using GraphPad software, as described in the figure legends.

## Results

### Screening of miRNAs targeting TfR mRNA 3′UTR

**Figure 1:**
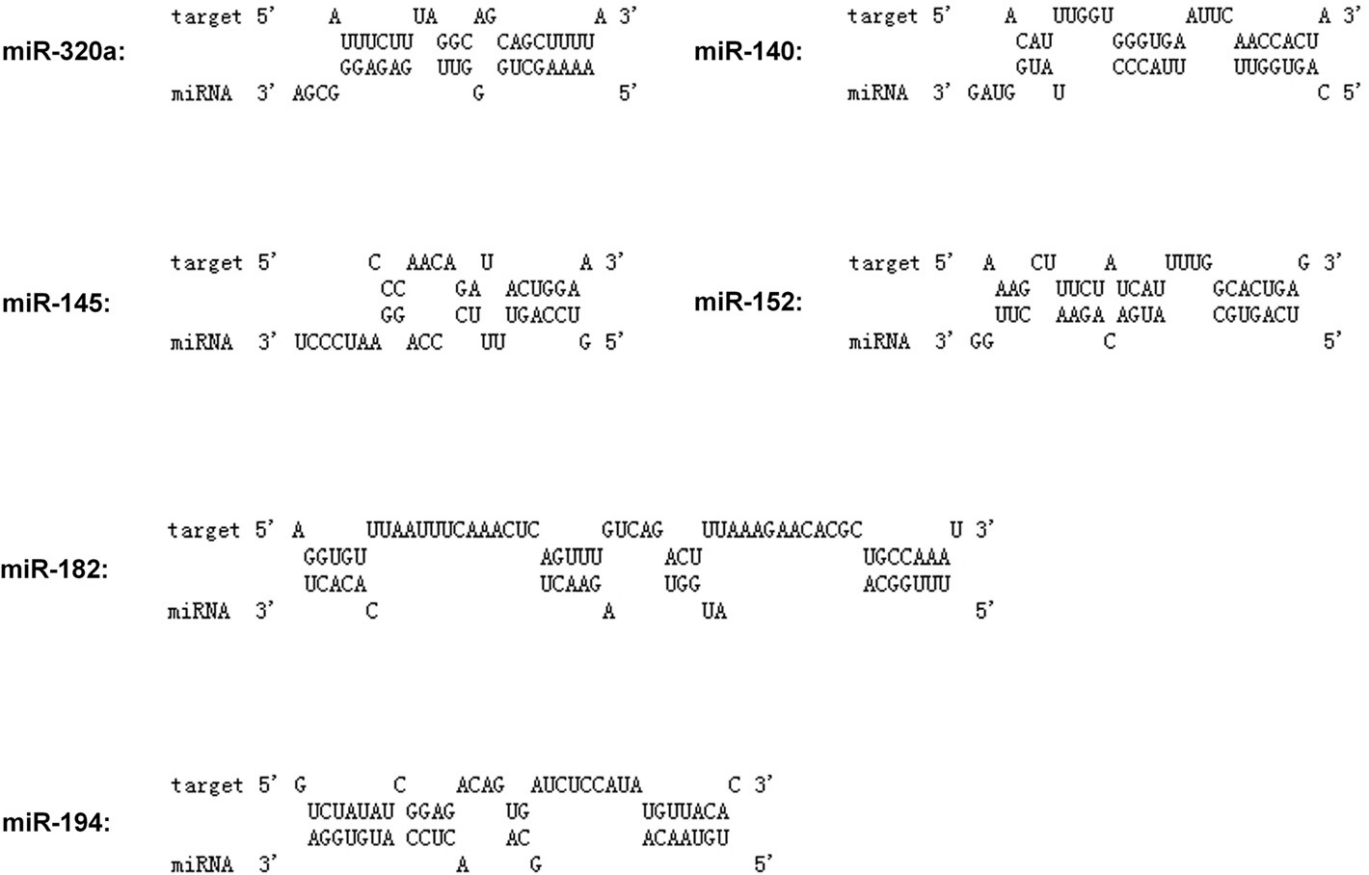
**Screening of miRNAs targeting TfR mRNA 3’UTR in F81 cells.** RNAhybrid and TargetScan tools were used to predict miRNA target sites on TfR mRNA 3′UTR following the rules of no mismatch and G/U complementary in miRNA seed sequences.

**Figure 2:**
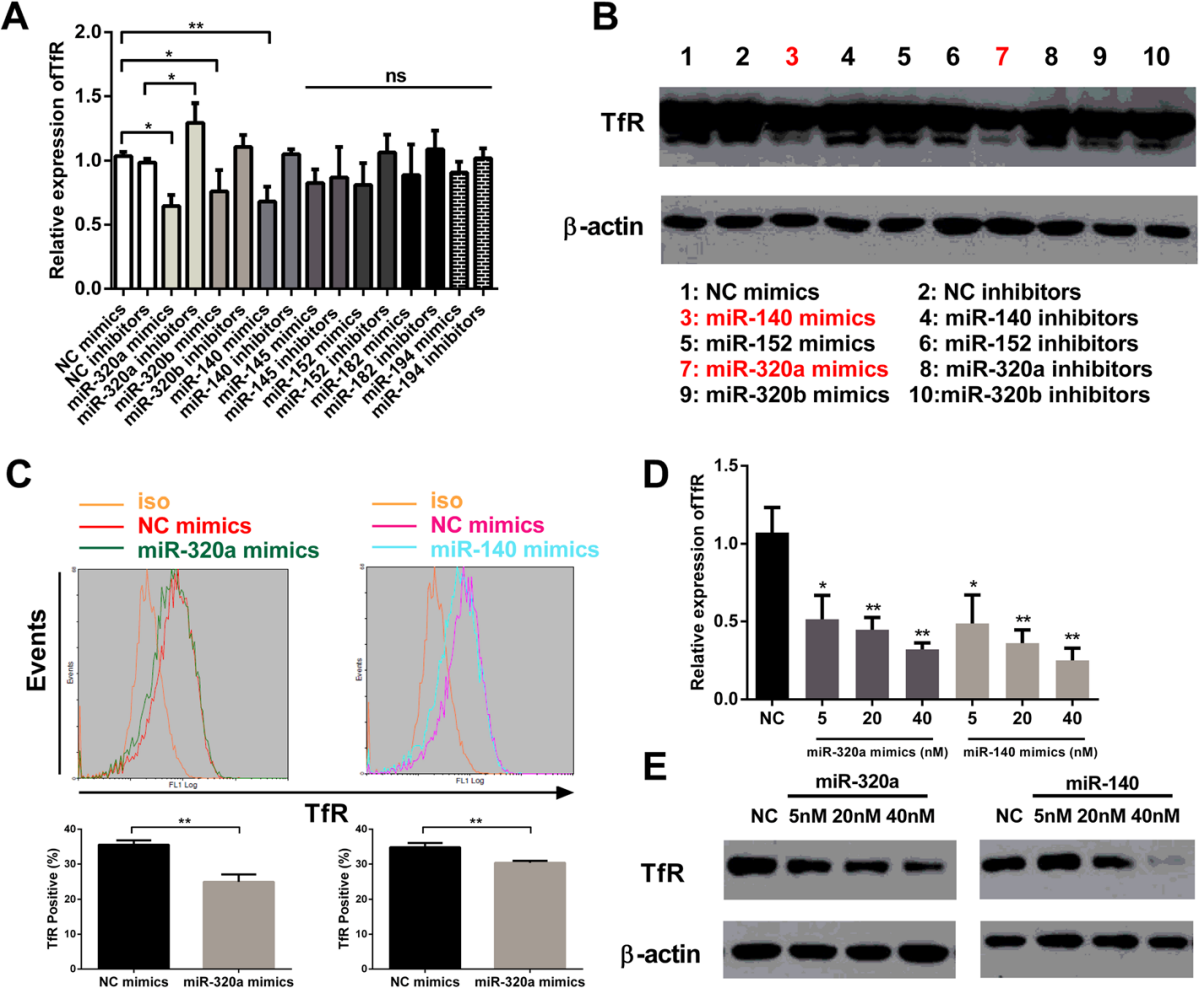
**MiR-320a and miR-140 inhibits TfR expression in F81 cells. (A)** qPCR was used to assess effects of predicted miRNA mimics and inhibitors on the relative expression of TfR after 36 h transfection with either mimics or inhibitors, with β-actin as an internal control. **(B)** Western blot assay was used to assess the TfR protein levels of lysates of F81 cells after 48 h transfection with either mimics or inhibitors. **(C)** Flow cytometric analysis was used to assess the percentage of TfR-positive F81 cells after 48 h transfection with miR-320a and miR-140 mimics. **(D)** qPCR was used to assess the TfR mRNA levels after 36 h transfection with miR-320a and miR-140 mimics in a dose-dependent manner **(E)** Western blot assay was used to assess the TfR protein levels after 48 h transfection with miR-320 and miR-140 mimics in a dose-dependent manner. Data are from 3 independent experiments (mean ± SD). Statistical significance was analyzed by Student’s *t* test; *P <0.05; **P <0.01; ns, not significant.

As described in Materials and Methods, small RNA ultrahigh throughput sequencing was performed (Solexa) on uninfected F81 cells and following MEV infection (MOI = 1) to detect miRNAs targeting TfR 3′UTR. Two miRNA libraries were also constructed [34]. Screening for miRNAs with RNAhybrid [30], RegRNA [31] and TargetScan tools identified 6 miRNA candidates (Figure 1). To test these miRNAs, F81 cells were transfected with the miRNA mimics and inhibitors, negative control (NC) mimics and inhibitors as controls. After 36 h, TfR mRNAs were quantified by qPCR. Results showed that miR-320a and miR140 mimics decreased TfR mRNA levels by almost 30% compared to NC mimics, and miR-320a inhibitors increased them approximately 25% (Figure 2A). After 48 h transfection as above, the quantity of TfR proteins were also examined by western blot and flow cytometry. Results showed that miR-320a and miR-140 downregulated the protein level (Figure 2B) and the percentage of TfR-positive cells by almost 20% and 30% respectively (Figure 2C). To further confirm the activity of the mimics, qPCR and western blot assay were used to show that the expression levels of TfR were reduced in a dose-dependent manner (Figure 1D,E). Altogether, these results clearly show that cellular miR-320a and miR-140 have negative effects on TfR expression.

### MEV infection leads to cellular miR-320a and miR-140 upregulation and TfR downregulation

**Figure 3:**
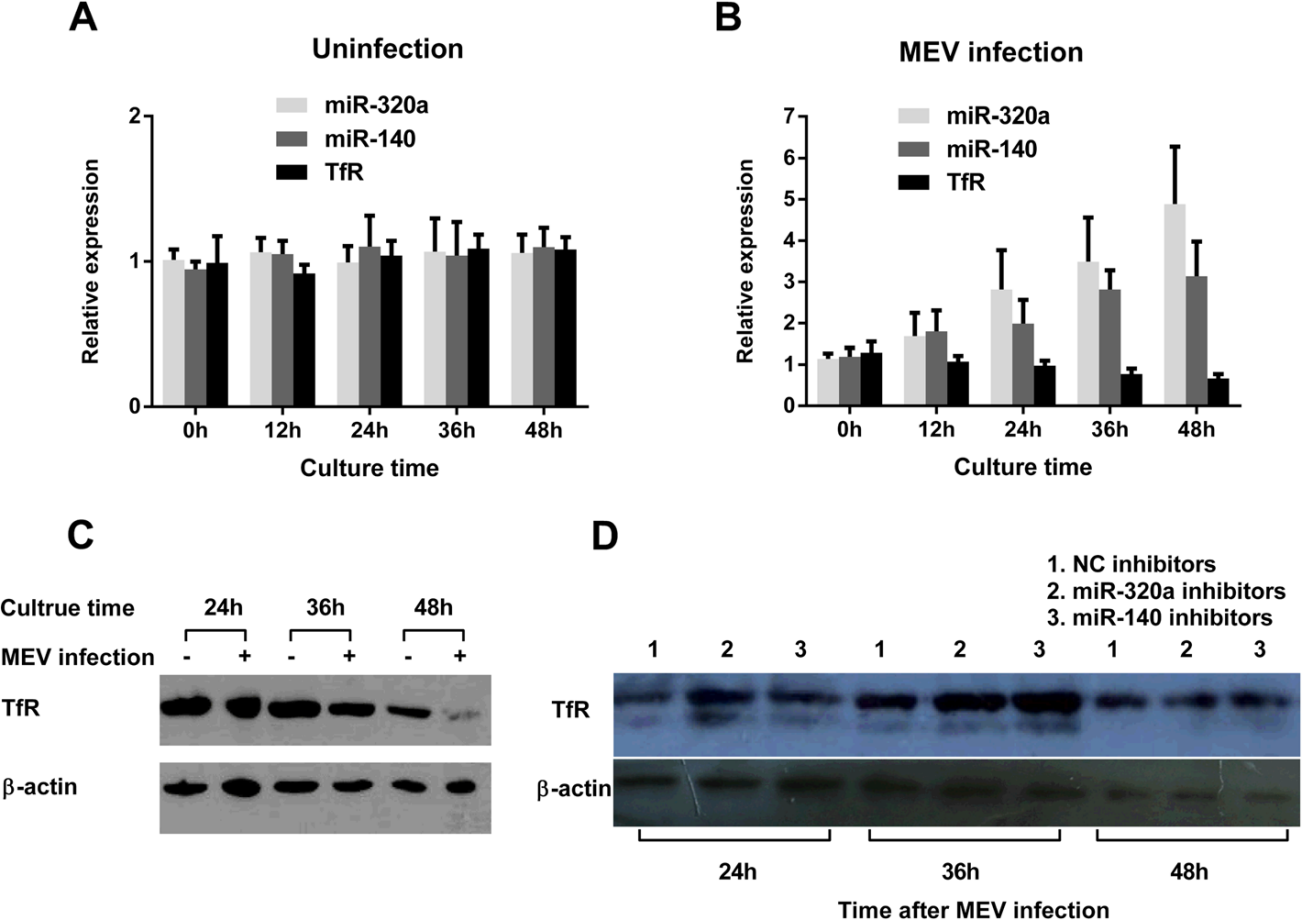
**MEV infection leads to gradual changes of miR-320a, miR-140 and TfR in F81 cells.** qPCR was used to assess the relative expression of miR-320a, miR-140 and TfR mRNAs at the indicated times **(A)** under normal conditions or **(B)** after MEV infection, with β-actin as an internal control. Data are from 3 independent experiments (mean ± SD). **(C)** Western blot assay was used to assess the TfR expression levels under normal conditions (−) or after MEV infection (+). **(D)** Western blot assay was used to assess the TfR expression levels when transfection with miRNA inhibitors post 12 h MEV infection.

The expression levels of predicted miRNAs are shown in Table 1. Following MEV infection both miR-320a and miR-140 showed relatively higher level expression and greater upregulation than found in uninfected cells. This was confirmed by qPCR which showed that both miRNAs gradually increased following MEV infection (Figure 3). To investigate whether upregulation of the two miRNAs following MEV infection could affect TfR expression, qPCR (Figure 3A,B) and western blot (Figure 3C) were performed simultaneously. As expected, TfR was gradually downregulated following MEV infection. To further ascertain that TfR downregulation is the result of miRNAs upregulation, F81 cells were transfected with miRNA inhibitors 12 h post MEV infection. Results showed that compared with transfection with NC inhibitors, both miR-320a and miR-140 inhibitors attenuated the inhibitory effect on TfR expression levels (Figure 3D). It appears, therefore, that upregulation of miR-320a and miR-140 directly results in TfR downregulation.

**Table 1:** **Expression level of the predicted miRNAs of the two libraries**

**miRNAs**	**Rds num** ^**a**^ **in mock infected cells**	**Rds num in MEV infected cells**	**TPM** ^**b**^ **in mock infected cells**	**TPM in MEV infected cells**
miR-320a	2432	64835	427.85	11566.02
miR-140	3005	8894	528.66	1586.61
miR-145	1181	668	207.77	119.17
miR-152	1208	561	212.52	100.08
miR-182	3094	5846	544.3	1042.88
miR-194	388	1	68.26	0.18

^a^Rds mum: Reads number.

^b^TPM: Tags per million.

### MiR-320a and miR-140 target the 3′UTR of TfR and physically bind to TfR mRNA in the RISC

**Figure 4:**
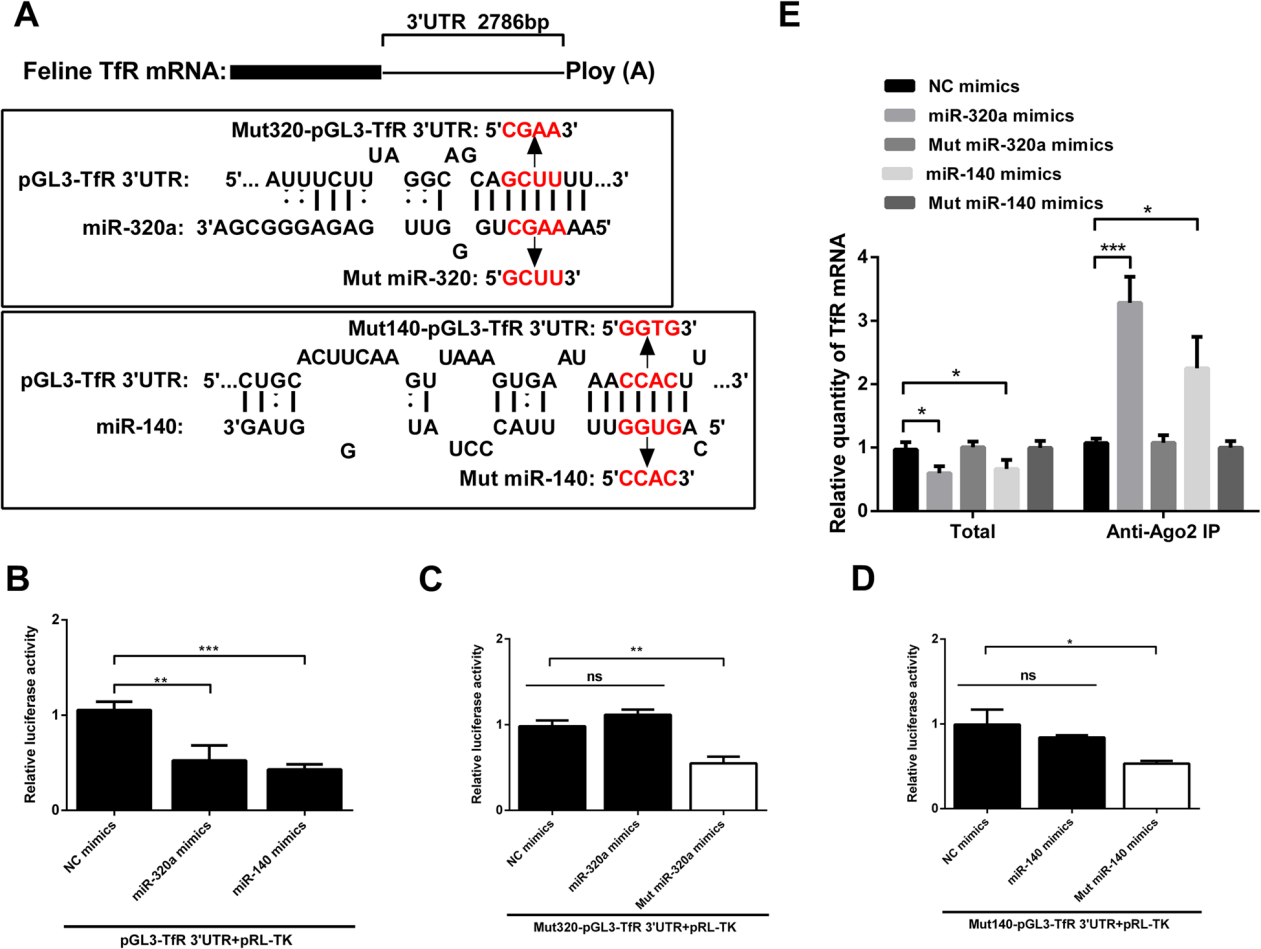
**MiR-320a and miR-140 target the 3′UTR of TfR and physically bind to TfR mRNA in the RISC. (A)** Schematic layout of TfR mRNA and presumptive targets of miR-320a and miR-140 via the sequential complementary nucleotides as indicated. The targets and tetranucleotide mutant targets segments were cloned into a pGL3-control reporter vector. The corresponding mut miR-320 and mut miR-140 were synthesized. **(B)** Luciferase activity of lysates of F81 cells after 36 h co-transfection with pGL3-TfR 3′UTR, pRL-TK and miR-320 or miR-140 mimics. NC mimics were used as controls. **(C)** Luciferase activity of lysates of F81 cells after 36 h co-transfection with mut320-pGL3-TfR 3′UTR, pRL-TK and miR-320a or mut miR-320a mimics. NC mimics were used as controls. **(D)** Luciferase activity of lysates of F81 cells after 36 h co-transfection with mut140-pGL3-TfR 3′UTR, pRL-TK and miR-140 or mut miR-140 mimics. NC mimics were used as controls. **(E)** qPCR analysis of TfR mRNA among RNAs extracted from Ago2 immunoprecipitates or total samples of F81 cells, normalized to β-actin. Data are from 3 independent experiments (mean ± SD). Statistical significance was analyzed by Student’s *t* test; *P <0.05; **P <0.01; ***P <0.001; ns, not significant.

To confirm that miR-320a and miR-140 directly target TfR 3′UTR and show inhibitory activity, a reporter vector pGL3-TfR 3′UTR containing the potential target segment in the 3′UTR of the luciferase gene was constructed (Figure 4A). After co-transfection of F81 cells with pGL3-TfR 3′UTR and miR-320a or miR-140 mimics, the relative luciferase activity was measured. As predicted, both miR-320a and miR-140 significantly inhibited the relative luciferase activity (by more than half) in comparison with NC mimics (Figure 4B). To further ascertain the necessary function of complementary seed sequence, mut320a-pGL3-TfR 3′UTR and mut140-pGL3-TfR 3′UTR, with mutated tetranucleotides were constructed and the corresponding mut miR-320a and mut miR-140 mimics were synthesized (Figure 4A). As expected, after co-transfection of F81 cells, miR-320a and miR-140 mimics had no effect on the relative expression activity of the mutant reporter vector, but mut miR-320a and mut miR-140 mimics downregulated them by more than 2-fold (Figure 4C*,*D). To provide evidence for the physical interaction of the two miRNAs with TfR mRNA, immunoprecipitation with Argonaute 2 (Ago2) was performed. As previously described [35], both mRNA degradation and translation repression is dependent on RISC, in which the most important factor is Ago2 protein. Therefore, anti-Ago2 antibody was used to test if the two miRNAs and TfR mRNA were associated with Ago2. As expected, when Ago2 protein was specifically precipitated by anti-Ago2 antibody, as compared with normal IgG, TfR was enriched more than 3-fold by miR-320a and more than 2-fold by miR-140 after transfection with miR-320a or miR-140 mimics respectively, whereas following transfection with mut miR-320a or mut miR-140, TfR was not enriched (Figure 4E). Altogether, these results indicate that miR-320a and miR-140 target the 3′UTR of TfR and physically bind to TfR mRNA in the RISC.

### MiR-320a and miR-140 inhibit MEV infection by preventing the virus from entering cells

**Figure 5:**
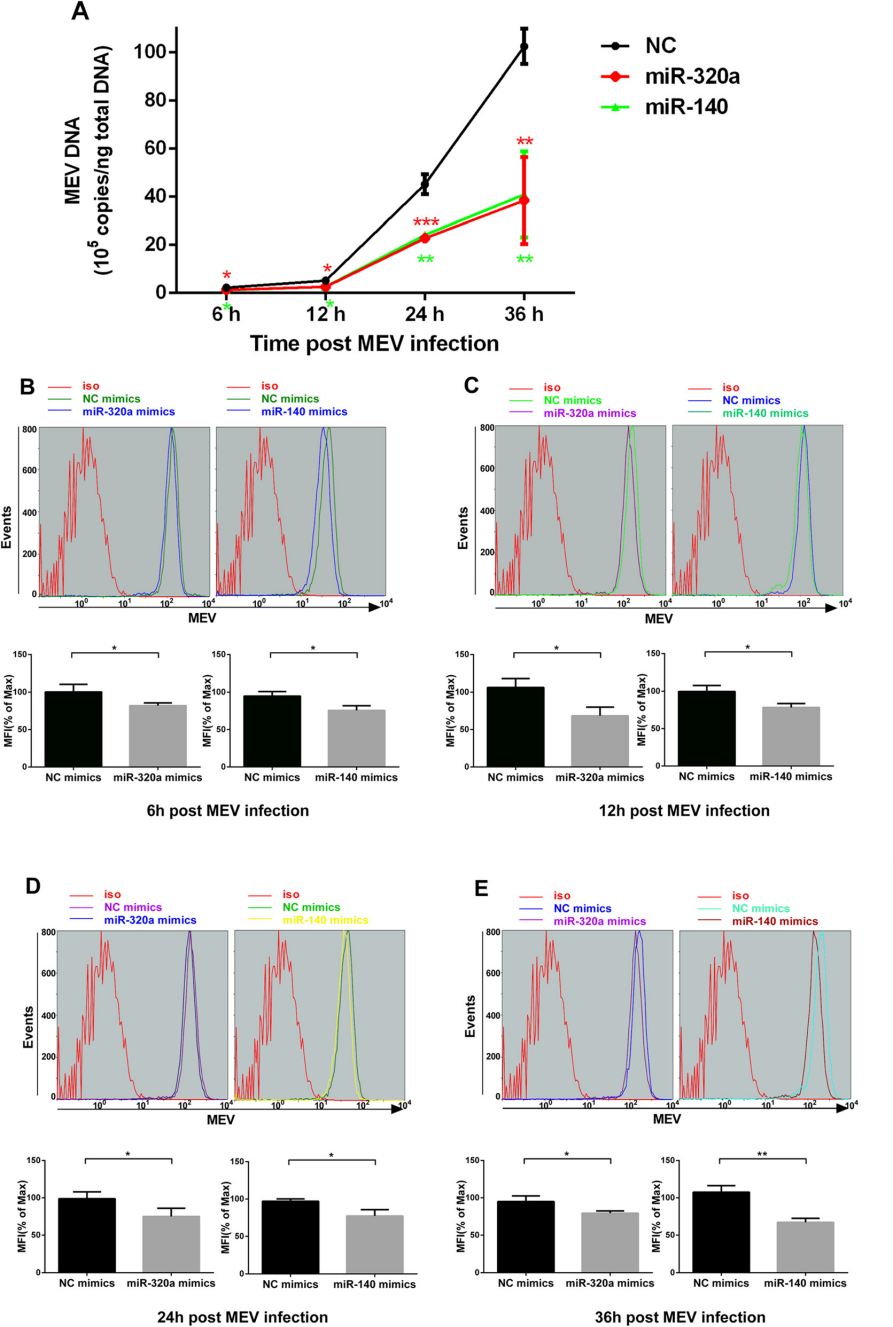
**MiR-320a and miR-140 inhibit MEV infection by preventing the virus to entry into F81 cells. (A)** qPCR was used to assess the effects of miR-320a and miR-140 mimics of MEV genomic DNA at the indicated times after 48 h transfection with the mimics. Data are from 3 independent experiments (mean ± SD). Statistical significance was analyzed by two-way ANOVA test; *P <0.05; **P <0.01; ***P <0.001. **(B,C,D,E)** Flow cytometric analysis was used to assess the effects of miR-320a and miR-140 mimics on MEV-infected F81 cells at the indicated times after 48 h transfection with the mimics. The mean fluorescence intensities (MFI) of MEV-infected F81 cells at the indicated times are shown. Data are from 3 independent experiments (mean ± SD). Statistical significance was analyzed by Student’s *t* test; *P <0.05; **P <0.01; ***P <0.001.

Since miR-320a and miR-140 downregulated TfR expression, we speculated that the two miRNAs could control MEV infection by preventing the virus from entering cells. To investigate this, F81 cells were transfected with either miR-320a and miR-140 mimics, with NC mimics as controls. After 48 h transfection, the cells were infected with MEV (MOI = 0.1). At the indicated times, the quantity of viral genomic DNA was measured. As predicted, results showed that miR-320a and miR-140 downregulated the quantity of viral genomic DNA in F81 cells, even during the early stage of virus infection (Figure 5A). To further validate the results above, flow cytometry was used to determine the proportion of MEV-infected cells. Results showed that compared with transfection with NC mimics, both miRNAs mimics had a negative effect on MEV-infected cell numbers from quite early on (Figure 5B,C,D,E). These results clearly demonstrated that miR-320a and miR-140 inhibit MEV infection by preventing virus entry into the cells.

### MiR-320a and miR-140 play roles on TfR and MEV in a synergistic manner

**Figure 6:**
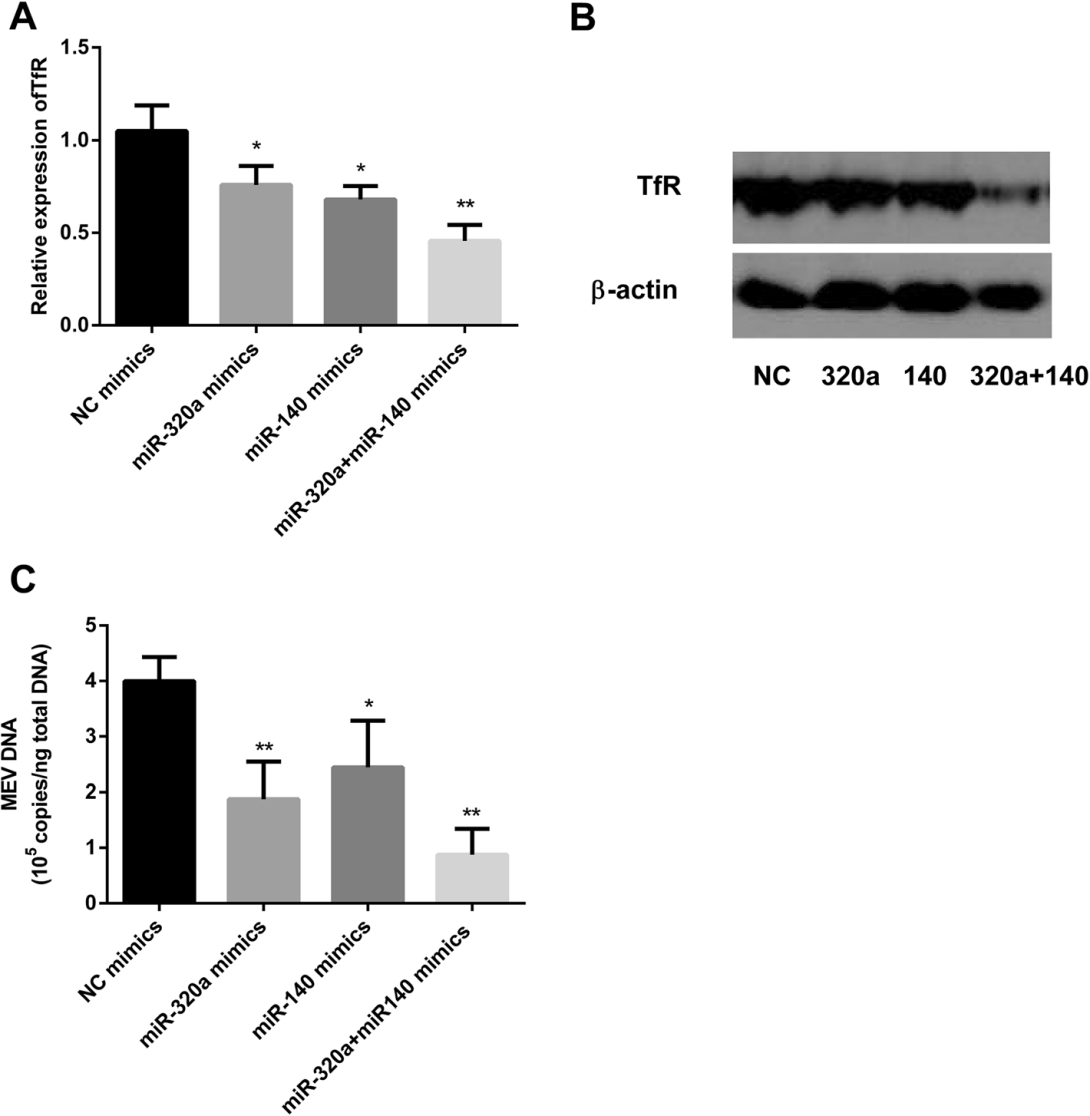
**MiR-320a and miR-140 play roles on TfR and MEV in a synergistic manner. (A)** qPCR was used to assess effects of the miRNAs on the relative expression of TfR after 36 h transfection with the mimics, with β-actin as an internal control. **(B)** Western blot assay was used to assess the TfR protein levels of lysates of F81 cells after 48 h transfection with the miRNAs mimics, with β-actin as an internal control. **(C)** qPCR was used to assess the effects of the miRNAs on MEV genomic DNA at 12 h post-MEV infection, after 48 h transfection with the mimics. Data are from 3 independent experiments (mean ± SD). Statistical significance was analyzed by Student’s *t* test; *P <0.05; **P <0.01.

As described above, both miR-320a and miR-140 inhibit MEV infection by downregulating TfR expression. Since the targets in TfR 3′UTR of the two miRNAs are different, we speculated that the two miRNAs could show function together. To investigate this, F81 cells were transfected with miR-320a and miR-140 mimics individually or together, with NC mimics as controls. After 36 h transfection, TfR mRNAs were assayed by qPCR. Results showed that miR-320a and miR140 mimics together decreased TfR mRNA levels by almost 30% compared to miR-320a or miR-140 mimics (Figure 6A). At 48 h, western blot assay also showed that the decrease in TfR protein level was enhanced by the two miRNAs together (Figure 6B). To confirm that the two miRNAs also showed greater negative function on MEV infection, F81 cells were transfected with miR-320a and miR-140 mimics individually or together, with NC mimics as controls. After 48 h transfection, the cells were infected with MEV (MOI = 0.1) and the viral genomic DNA measured 12 h later. As predicted, results showed that the two miRNAs together produced a greater reduction in viral genomic DNA than individually.

## Discussion

Host cellular miRNAs have frequently been reported to interact with viruses during infection [10,15,35-40]. We recently showed that miR-181b inhibited MEV replication by repression of its non-structural protein 1 expression [18]. Here, we report that other miRNAs, miR-320a and miR-140, inhibit MEV entry into F81 cells by downregulating its receptor, TfR, through targeting the 3′UTR of TfR mRNA.

A number of reports have shown that TfR, as a cell surface receptor, is required for iron delivery to cells. Indeed, TfR has been established as a gatekeeper for regulating iron uptake by most cells, and the transferring-to-cell endocytic pathway has been characterized in detail [41]. TfR is central to the uptake of iron-loaded transferrin [42] which is post-transcriptionally regulated via iron-responsive elements present in its 3′UTR [43]. In addition to its role in erythropoiesis, TfR is also overexpressed in the majority of malignancies [44] and is directly linked to cell proliferation [45-47]. Studies have demonstrated that parvovirus replication is dependent on host cellular division and proliferation ([48]; Tattersa [49]) and vigorous proliferative activity of host cells promotes parvoviral replication. Reduction in cellular metabolic activity, therefore, may explain why downregulation of TfR inhibits MEV replication. There may, however, be another mechanism. TfR is necessary for MEV uptake by host cells: downregulation may therefore render infected cells less susceptible to superinfection with additional MEV particles. While it may take only one virion to establish a primary infection, a multiply-infected cell may produce more infectious progeny. Downregulation of TfR on the cell surface, therefore, may result in diminished production of virus.

**Figure 7:**
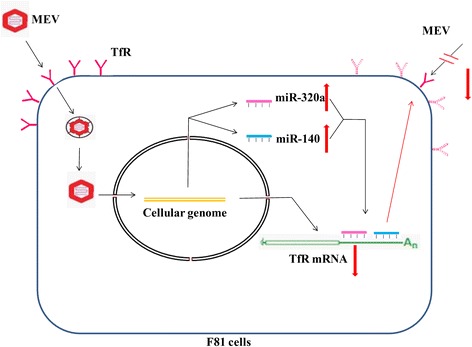
**Schematic layout of the mechanism of interaction between MEV and its host F81 cells involving two miRNAs and TfR.** (Up arrows: upregulation; down arrows: downregulation).

As summarized in Figure 7, MEV infection leads to increase in two host cell miRNAs (miR-320a and miR-140) which downregulate TfR expression, resulting in a decrease in viral replication. Since TfR is a specific receptor of MEV, however, reduction in it may prevent cellular entry of further virus following the primary infection, resulting in protection of host cells from immediate death as a result of continuing virus infection. Although two host cell miRNAs were found to be upregulated by MEV infection, the mechanism remains unclear. We have identified one mechanism of interaction between MEV and its host F81 cells, however, that might explain why a cell can be infected with just one virus.

Since miR-320a and miR-140 have been shown to inhibit MEV entry into host cells and might also affect virus replication, it may find application as an antiviral therapeutic for MEV-induced mink enteritis. As several reports have shown [50-54], siRNAs can be used to control virus diseases *in vivo*; however, little attention has so far been paid to the possibility of using endogenous miRNAs as an antivirus tool. Compared with siRNAs, endogenous miRNAs may be safer and induce fewer side-effects. More extensive studies are merited to determine if the two miRNAs described here can be used as antiviral tools.

## Conclusions

In conclusion, our work has shown that two miRNAs (miR-320a and miR-140) inhibit MEV entry into the F81 cells by downregulating its specific receptor TfR through targeting the 3′UTR of TfR mRNA in a synergistic manner, while the two miRNAs were upregulated through MEV infection. As summarized in Figure 7, a simple pathway of host-virus interaction network involving TfR and miRNAs has been deduced. These results provide further understanding of the mechanisms in MEV infection and may be helpful for development of endogenous miRNA antiviral therapy strategies.
